# FZR1 as a novel biomarker for breast cancer neoadjuvant chemotherapy prediction

**DOI:** 10.1038/s41419-020-03004-9

**Published:** 2020-09-25

**Authors:** Shuo Liu, Haobin Wang, Jun Li, Jianhui Zhang, Jian Wu, Yi Li, Yongjun Piao, Leiting Pan, Rong Xiang, Shijing Yue

**Affiliations:** 1 School of Medicine, Nankai University, Tianjin, China; 2 Department of Breast & Thyroid Surgery, The Third People’s Hospital of Chengdu, The Affiliated Hospital of Southwest Jiaotong University, The Second Chengdu Hospital Affiliated to Chongqing Medical University, Chengdu, Sichuan China; 3 Sichuan hospital & Institute, Sichuan cancer center, School of Medicine, University of Electronic Science and Technology of China, Chengdu, Sichuan China; 4 Department of Radiology, The Third People’s Hospital of Chengdu, The Affiliated Hospital of Southwest Jiaotong University, The Second Chengdu Hospital Affiliated to Chongqing Medical University, Chengdu, Sichuan China; 5 The Key Laboratory of Weak-Light Nonlinear Photonics of Education Ministry, School of Physics and TEDA Institute of Applied Physics, Nankai University, Tianjin, China; 6 2011 Project Collaborative Innovation Center for Biotherapy of Ministry of Education, Tianjin, China

**Keywords:** Breast cancer, Predictive markers, Breast cancer

## Abstract

The concept of breast-conserving surgery is a remarkable achievement of breast cancer therapy. Neoadjuvant chemotherapy is being used increasingly to shrink the tumor prior to surgery. Neoadjuvant chemotherapy is reducing the tumor size to make the surgery with less damaging to surrounding tissue and downstage locally inoperable disease to operable. However, non-effective neoadjuvant chemotherapy could increase the risks of delaying surgery, develop unresectable disease and metastatic tumor spread. The biomarkers for predicting the neoadjuvant chemotherapy effect are scarce in breast cancer treatment. In this study, we identified that FZR1 can be a novel biomarker for breast cancer neoadjuvant chemotherapy according to clinical patient cohort evaluation and molecular mechanism investigation. Transcriptomic data analysis indicated that the expression of FZR1 is correlated with the effect of neoadjuvant chemotherapy. Mechanistically, we demonstrate that FZR1 is pivotal to the chemotherapy drugs induced apoptosis and cell cycle arrest. FZR1 is involved in the stability of p53 by impairing the phosphorylation at ser15 site. We demonstrate that the expression of FZR1 detected by quantification of IHC can be an effective predictor of neoadjuvant chemotherapy in animal experiment and clinical patient cohort. To obtain more benefit for breast cancer patient, we propose that the FZR1 IHC score using at the clinical to predict the effect of neoadjuvant chemotherapy.

## Introduction

To date, breast cancer is considered as a heterogeneous disease with distinct subtypes based on the gene expression profiling. In the field of breast cancer treatment, a comprehensive treatment strategy is developed that included surgery, chemotherapy, radiotherapy, endocrine therapy and molecular targeted therapy^1,2^. Surgical treatment is still the most important approach for breast cancer. The concept of breast-conserving and the development of breast-conserving surgery is one of the most remarkable achievements of cancer therapy. Currently, Neoadjuvant chemotherapy (NACT) contributes to the idea of breast-conserving cancer therapy that chemotherapy drugs are delivered before surgery to shrink the tumor. NACT is reducing the tumor size prior to surgery makes the surgeon with less damage to surrounding tissue and enables the surgeon to better differentiate the edge of the tumor from healthy tissue^3^. NACT also reduces the inflammation of neighboring metastasis tissue and allows more of the healthy tissue to remain. Overall, NACT improves the outcome of breast cancer treated with surgery^4,5^. It is suppose that the combination of NACT with traditional treatment will bring the best benefits to patients in the research field^6^.

NACT is widely used in breast cancer to downstage locally advanced (inoperable) disease and make it operable, particularly for large tumors^7,8^. There is a number of potential advantages of NACT, including reduction of tumor burden to facilitate surgical resection, insights into the chemotherapy drugs resistance and response, important information of prognostic, toxicity, and survival^9,10^. Studies of preclinical models suggest that NACT might provide a survival benefit over adjuvant therapy in the context of treatment with an immune checkpoint blockade^11,12^. Although the listed potential benefits of NACT, the risks of delaying surgery with poor treatment response could develop unresectable disease and increased chances for metastatic tumor spread. In addition, the toxic effects of NACT might aggravate morbidity or further delay surgical resection, and increasing surgical risk^13,14^. Thereby, it is greatly need to develop a specific biomarker signature to define the ideal patient population for NACT. However, the molecular biomarkers are scarcity for the pathological response of NACT in breast cancer.

Recent studies demonstrate that the efficacy of NACT differs significantly among breast cancer subtypes and the NACT ability to increase the risk of cancer progression. The increasing evidence concluded that NACT elevated the local recurrence rate, despite reduced tumor size^9,15^. An unfavorable effect of chemotherapy is associated with the selection of chemoresistant clones and the conditions for cancer progression^16^. It is believed that NACT promotes metastasis via the induction of cellular stress and remodeling tumor microenvironment^17–20^. According to the current knowledge, we need to establish a consensus and recommendations for the NACT in breast cancer. Investigations to identify the biomarker or the methods for the efficacy of NACT have been executed in breast cancer^21–23^. Although these biomarkers are considered to be the predictor of pathologic complete responses to NACT in breast cancer, there is a large limitation for the clinical application. The existing biomarkers for NACT have been reported including the lncRNA profile, the change of axillary lymph nodes, and the recurrence scores with NACT. The lncRNA profiling has identified several lncRNAs associated with the pathological complete response, which is limited for clinical application. The change of axillary lymph nodes and the recurrence scores can be explored during or proceed the NACT that are not suitable for clinical application.

In this study, we identified that FZR1 as a biomarker for breast cancer NACT based on the transcriptomic data analysis and the molecular mechanism investigation. FZR1 is involved in the regulation of the stability and transcriptional activity of tumor suppressor p53. The molecular function of FZR1 to modulate the cell apoptosis and cell cycle arrest by chemotherapy drug induction. The validation with a cohort of clinical patient samples demonstrated that the expression of FZR1 can be a biomarker for the efficacy of NACT. The evaluation was performed by the IHC and the quantification of optical density score, which is feasible and suitable for the clinical application.

## Materials and methods

### Cell lines and culture

Human breast cancer cells T-47D and MDA-MB-231 were purchased from ATCC. T-47D cells were cultivated in RPMI-1640 medium supplemented with 10% fetal bovine serum (FBS) and 1% penicillin/streptomycin. MDA-MB-231 cells were maintained in Dulbecco’s modified Eagle’s medium (DMEM) supplemented with 10% fetal bovine serum (FBS) and 1% penicillin/streptomycin. HEK-293T cells were purchased from ATCC and maintained in Dulbecco’s modified Eagle’s medium (DMEM) supplemented with 10% FBS and 1% penicillin/streptomycin.

### Patient derived samples

Breast cancer clinical samples were obtained from The Third People’s Hospital of Chengdu and Sichuan Cancer Center. This study was approved by the institutional ethics committees of The Third People’s Hospital of Chengdu. All patients signed an informed consent. Patient’s information is reported in Table 1.

**Table 1:** Clinical information of breast cancer patients for neoadjuvant chemotherapy.

Characteristics (*N*)	Luminal A (14)	Luminal B (122)	Her-2 (45)	Basal (12)	*P*-value
Median age at diagnosis (range), year	37, (33–69)	57, (22–78)	43, (30–72)	45, (32–77)	0.5785
Gender, *N* (%)					0.6207
Female	14	119	45	12	
Male	0	3	0	0	
Neoadjuvant chemotherapy type, *N* (%)					0.5242
TEC	14	110	40	10	
Others	0	12	5	2	
Neoadjuvant chemotherapy cycle					0.2307
1	2	6	1	0	
2	3	24	4	2	
3	3	23	6	3	
4	3	18	15	2	
5	1	13	4	0	
6	0	11	4	4	
7	1	2	2	0	
8	2	22	7	1	

### Plasmids and transfections

The human FZR1 and CHK2 cDNA derived from the breast cancer cell line T-47D fused with Flag, His or Myc-peptide at the C-terminal was cloned into the lentiviral expression vector pLV-EF1α-MCS-IRES-Bsd/puro (Biosettia, San Diego, CA, USA). The TP53 shRNAs were cloned into the pLV-RNAi-Vector (Biosettia, San Diego, CA, USA). The FZR1 sgRNA was cloned into the lentiCRISPRv2 vector (Addgene). All constructs were verified by DNA sequencing. The primers used for the generation of gene overexpression, shRNAs for gene knockdown and sgRNA for gene knockout cell lines are listed in Supplementary Table 1.

### Transduction of lentivirus

Stable breast cancer cell lines expressing FZR1-Flag, sgRNA or shRNA were established using a lentiviral-based delivery system. For the generation of lentiviral particles, 4 × 10^6^ HEK293T cells were seeded in a 10 cm culture dish and cultivated overnight. Next day cells were co-transfected with 9 µg of empty or FZR1-containing pLV-EF1α-MCS-IRES-Bsd/puro vector, or shRNA-containing pLV-RNAi-Vector, or sgRNA-containing lentiCRISPRv2 plasmids together with packaging and envelope vectors (4.5 µg pMDLg/pRRE, 1.8 µg pRSV-REV, 2.7 µg pCMV-VSV-G purchased from Addgene) using Lipofectamine 2000 (Invitrogen). Transfection was performed following manufacturer’s recommendations. Culture medium containing lentiviral particles was collected 48 h post-transfection and filtered through a 0.45 µm membrane. Lentiviral particles were diluted 1:2 in fresh medium and supplemented with 8 μg/ml polybrene. For lentiviral infection, target cells were incubated with lentiviral particles for 6–8 h. After incubation, cells were supplemented with fresh medium and incubated for an additional 48 h. Stable cells were selected using puromycin (1 µg/ml). All the viral experiments were performed in a biological safety cabinet.

### Quantitative real-time PCR (qRT-PCR) and RNAseq

Total RNA was extracted using TRIZOL reagent (Invitrogen) following manufacturer’s instructions. cDNA was then synthesized using the Trans Script First-Strand cDNA Synthesis Super Mix Kit (Trans Gen Biotech, Beijing, China). qPCR was performed in a CFXTM real-time thermal cycler (Bio-Rad, Hercules, CA, USA) using a Trans Start Top Green qPCR Supper Mix kit (Trans Gen Biotech). Data analysis was performed with the comparative ΔCt method using GAPDH as internal control^24^. The sequences of the primers used in this study are listed in Supplementary Table 2.

Total RNA was performed the sequencing on a BGISEQ-500 platform by BGI group. Raw reads were aligned to transcriptome using Bowtie2 (v.2.5.5) and RESM using a human hg38 reference from UCSC.

### Western blot analysis and immunoprecipitation

Adherent cells were washed twice with cold PBS and collected by scraping. Cellular pellets were re-suspended in RIPA lysis buffer (25 mM Tris-HCl pH 7.6, 150 mM NaCl, 1% sodium deoxycholate, 0.1% SDS) supplemented with protease and phosphatase inhibitor cocktail (Sigma‐Aldrich, St. Louis, MO, USA) and incubated on ice for 30 min. Protein lysates were cleared by centrifugation (16,200 *g* for 15 min at 4 °C) and the protein concentration was assessed by BCA Protein Assay (Bio-Rad). Proteins were separated onto polyacrylamide gels and transferred to PVDF membranes. Membrane was blocked for 1 h in 5% milk in TBS-T (w/v) and incubated overnight at 4 °C with primary antibodies on a shaking platform. On the next day, membranes were washed 5 times in TBS-T and incubated with appropriate HRP-conjugated secondary antibodies for 1 h. After incubation, membranes were extensively washed in TBS-T prior to signal detection using the Tanon Chemiluminescent Imaging System (Tanon, Shanghai, China). The primary antibodies used in this study are listed in Supplementary Table 3. For immunoprecipitation, cell lysates were collected in IP buffer (50 mM Tris-HCl; pH 7.4, 150 mM NaCl, 0.1% NP-40, 5 mM EDTA) supplemented with protease inhibitor cocktail (Sigma-Aldrich) and cleared by centrifugation. Protein lysates were incubated with specific antibodies as indicated in the figure legends or with non-immune immunoglobulin (IgG; negative control) and with Protein G-agarose beads (GE Healthcare) overnight on a rotating wheel. Next day, the beads were washed in ice-cold IP buffer, resuspended in 2× western blot loading buffer (65 mM Tris-HCl pH 6.8, 25% glycerol, 2% SDS, 0.01 % bromophenol blue, 50 mM DTT) and boiled at 95 °C for 5 min prior to western blot analysis.

### Immunofluorescence

Cells were seeded onto coverslips, fixed with 100% methanol for 15 min at room temperature and washed twice in ice-cold PBS. Cells were blocked in 5% goat serum in PBS at room temperature for 1 h and incubated overnight at 4 °C in primary antibody. Then cells were washed 3 times in PBS and incubated with Alexa Fluor 488 or Alexa Fluor 594 conjugated secondary antibodies (ZSGB-BIO, Beijing, China) for 1 h at room temperature. Cells were counterstained with DAPI. Pictures were captured using a confocal microscope (Olympus, Tokyo, Japan).

### Immunohistochemistry (IHC)

Mice or human tissue paraffin sections were dewaxed and rehydrated, followed by antigen retrieval by boiling sections with 0.01 M citrate buffer (pH 6.0) and washed twice with PBS. The endogenous peroxidase was quenched by immersing section into 3% hydrogen peroxide for 10 min and washed twice with PBS. The sections were incubated with 5% goat serum at room temperature for 45 min and then incubated with primary antibody overnight at 4 °C. Next day, samples were incubated with HRP-ligated Goat anti-rabbit/mouse IgG (ZSJQ-BIO) and diaminobenzidine (DAB) was used as a sensitive chromogen, and then nucleus were counterstained with hematoxylin. Images acquired using an optical microscope (Olympus, Tokyo, Japan). Hematoxylin and eosin (H&E) staining was performed using standard procedures.

### Chemotherapeutic drugs sensitivity assay

1 × 10^4^ cells were seeded onto 96 well plate and treated with various concentrations of cisplatin, epirubicin or doxorubicin for 24 or 48 h h. The cell viability was determined using a Cell Counting Kit-8 (Dojindo, Kumamoto, Japan). Cell counting was performed following manufacturer’s instructions and IC50 of the chemotherapeutic drugs was calculated using Graphpad software.

### Super-resolution microscopy analysis

Cells were fixed with pre-cooled methanol on coverslips and performed immunofluorescence staining. Fluorescence images of single molecules were recorded and super-localized over 50000 camera frames^25^. Two-dimensional cross-correlation analysis was performed by calculating the pairwise intermolecular distances between individual molecules identified in two color channels^26,27^. The distance distribution was normalized by the results of multiple sets of molecules randomly distributed in the same region. Therefore, the normalized cross-correlation amplitudes obtained by shifting the position indicate the correlation and non-correlation of the values of the two color channels >1 and <1, respectively.

### Annexin-V-FITC/PI staining

Annexin-V-FITC/PI staining was performed using Annexin-V-FITC Apoptosis Detection Kit (Thermo Fisher Scientific) and following the manufacturer’s instructions. Briefly, cells were resuspended in binding buffer. Annexin-V-FITC reagent and PI were added into 100 μl cell solution (∼1.0 × 10^5^ cells) cell solution and incubated for 15 min in the dark. Each sample was added 400 μl binding buffer and analyzed by flow cytometry with BD FACScan system (BD Biosciences).

### Animal models

BALB/c nude (nu/nu) mice (6-week old, Female) were purchased from Vital River Laboratory Animal Technology Co. Ltd (Beijing, China) and maintained in pathogen-free facility of Nankai University. All of the animal experiments were approved by the Nankai University Animal Care and Use Committee and handled according to the Nankai University Animal Welfare Guidelines. For the mice xenograft model, 2 × 10^6^ T-47D cells stably knockout FZR1 or a control empty vector were subcutaneously injected into 6 week-old female nude mice (*n* = 4 per group). Cisplatin was administered by intraperitoneal injection from week 2 post cell injection for 5 mg/kg body weight once a week and for 4 weeks. Tumor volume was measured starting from week 3 post-injection for twice every week and calculated using the standard equation: *V* = 1/2 × *L* × *W*^2^, where *V* is the tumor volume, *L* is the tumor length and *W* is the tumor width.

### TUNEL assay

TUNEL staining was performed by using of DeadEnd™ Fluorometric TUNEL System (Promega, G3250) according to the operating manual. Briefly, paraffin sections were dewaxed and rehydrated with xylene and graded alcohol, followed by immersion into distilled water. After two wash with PBS, sections were permeabilized by incubated with 20 µg/ml Proteinase K at room temperature for 10 min, then the slides were washed twice with PBS. After equilibration with EB buffer, reaction solution was added to cover the tissues and the slides were placed in a humidified chamber, incubated for 60 min at 37 °C. Reaction was terminated by immersing slides in 2X SSC solution for 15 min, followed by two wash with PBS, then the slides were mounted with DAPI-Fluoromount G (Southern Biotech, 0100-01).

### Statistical analysis

Data are expressed as the average ± standard deviation of at least 3 independent experiments. Statistical significance was assessed using the Student’s *t*-test and GraphPad Prism 5 software (Inc., La Jolla, CA).

## Results

### Identification of FZR1 as a potential biomarker for NACT in breast cancer

**Fig. 1:**
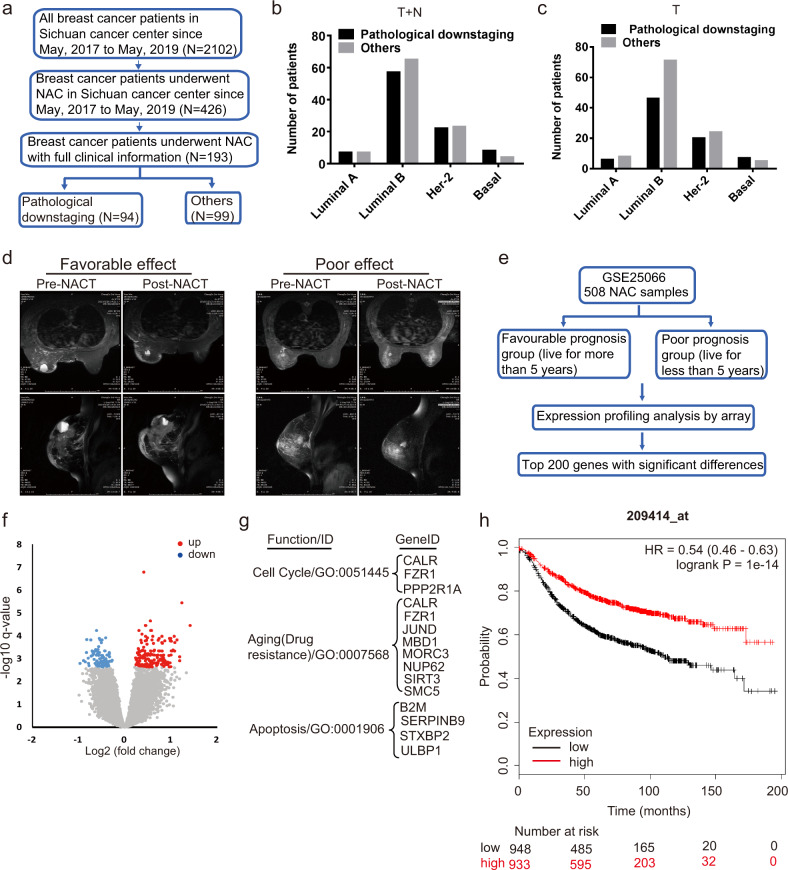
Identification of the potential biomarker for breast cancer neoadjuvant chemotherapy. **a** Identification of patients with breast cancer who underwent neoadjuvant chemotherapy as primary treatment in the Sichuan cancer center in China between 2017 and 2019. **b**, **c** Clinical evaluation of pathological downstaging of T (tumor) and TN (tumor, Lymph Node) stages based on molecular classification. **d** A set of representative MRI images of the favorable and poor effect of NACT for breast cancer treatment is shown. **e** Data analysis and mining from the data resources GSE25066 to find the relevant genes of neoadjuvant chemotherapy effect. **f**, **g** Gene expression analysis and gene ontology enrichment were performed to find the top relevant genes of neoadjuvant chemotherapy effect. **h** Survival analysis of breast cancer patients with neoadjuvant chemotherapy to the expression of FZR1 in the database of KMPLOT.

To identify a biomarker for NACT, we analyzed a breast cancer cohort that patients with clinical T1–T4 were diagnosed and received NACT with a full recorded tumor grade between 2017 and 2019 in Sichuan cancer center (Fig. 1a). Patients were grouped into the favorable and poor effect of NACT according to the clinical diagnosis and the NACT cycles. The general patient characteristics of cohort are shown in Table 1. Patients with clinical T grade pathological downstage analysis, luminal A had 7 (50%), luminal B had 57 (47%), Her2 had 22 (49%), and Basal had 8 (67%) favorable effect of NACT (Fig. 1b). Patients with clinical T and N grade pathological downstage analysis, luminal A had 6 (46%), luminal B had 46 (39%), Her2 had 20 (45%), and Basal had 7 (58%) favorable effect of NACT (Fig. 1c). A representative MRI imaging prior to and post NACT for the favorable or poor effect is shown (Fig. 1d).

To gain insight into the gene expression profiles relevant to the effect of NACT, we screened the publicly available transcriptomic data, including GEO (GSE25055, GSE25065, GSE25066) and The Cancer Genome Atlas (TCGA), focusing on the significant difference in favorable and poor prognosis groups (Fig. 1e, f)^5^. Top 200 genes with significant difference correlated to the effect of chemotherapy were further analyed with gene ontology. The thirteen genes were enriched in the function of cell cycle, drug resistance, and apoptosis regulation (Fig. 1g). Moreover, the relationship on the expression of the thirteen genes and patient progression-free survival were analyzed using online tool Kaplan–Meier Plotter (http://kmplot.com/analysis/) (Supplementary Fig. S1). FZR1 was identified as a potential candidate biomarker for the effect of NACT (Fig. 1h).

**Fig. 2:**
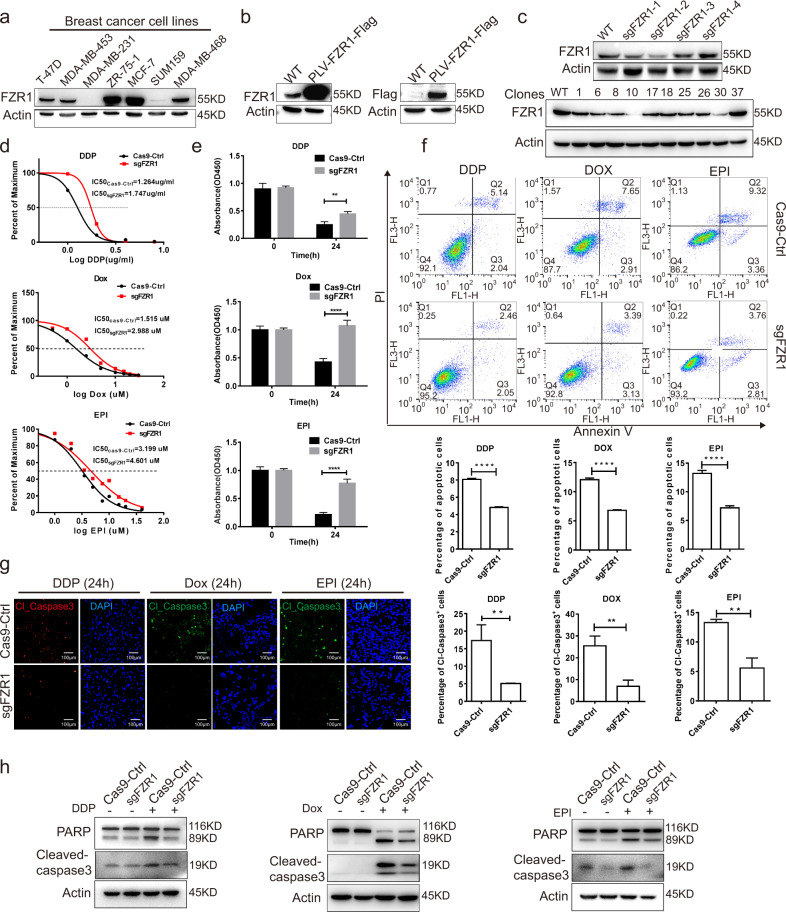
FZR1 is involved in the chemotherapy drug induced apoptosis. **a** The expression of FZR1 in the variety of breast cancer cell lines. A representative western blot of three independent experiments is shown. **b** FZR1 fuse Flag tag overexpression T-47D stable cell line was generated by lentiviral transduction. **c** FZR1 knockout (ko) T-47D cell line was generated by CRISPR/Cas9 technique and single cell cloning. **d** FZR1 ko increased the IC 50 of T-47D cells to the chemotherapy drugs cisplatin, doxorubicin and epirubicin. **e** FZR1 ko increased the cell viability of T-47D to the chemotherapy drugs cisplatin, doxorubicin and epirubicin treatment. The graph represents the average of cell staining absorbance ± SD of 3 different times. **p < 0.01, ****p < 0.001. **f** Apoptosis analysis by Annexin-V/PI double staining. Annexin-V/PI double staining was performed on control and FZR1 ko T-47D cells treated with 2 µg/ml cisplatin, doxorubicin, and epirubicin for 12 h. The quantification are representative of experiments in triplicate, and the percentages of apoptotic cells are shown in the relevant quadrants ****p < 0.001. **g** IF of cleavage caspase 3 was performed in control and FZR1 ko T-47D cells treated with 2 µg/ml cisplatin, doxorubicin, and epirubicin for 24 h. The quantification are representative of the 10 fields ± SD by random **p < 0.01; scale bar 100 µm. **h** FZR1 ko reduced the cleavage of apoptotic protein PARP and caspase 3 level. A representative western blot of three independent experiments is shown.

To further investigate the functional role of FZR1 in chemotherapy sensitivity, the endogenous levels of FZR1 in various breast cancer cell lines were explored by western blot (Fig. 2a). We revealed that the expression of FZR1 is increased with the chemotherapy drugs treatment by qPCR and western blot (Supplementary Fig. S2a–c). The stable T-47D cells overexpressed FZR1 fused Flag tag protein was generated by lentiviral infection. FZR1 knockout (ko) stable T-47D cell line was created using CRISPR/Cas9 system and single cell screening. As expected, FZR1 overexpression and knockout were confirmed by western blot (Fig. 2b, c). FZR1 ko significantly increased the IC50 of the first-line chemotherapy drugs including cisplatin, doxorubicin, and epirubicin in T-47D cells (Fig. 2d). The CCK8 assay indicated that FZR1 ko significantly suppressed the chemotherapy drugs induced apoptosis in T-47D cells (Fig. 2e). We also confirmed that overexpression FZR1 promotes chemotherapy drug induced apoptosis in MBA-MD-231 breast cancer cell line (Supplementary Fig. S3a, b). FZR1 ko significantly inhibited the chemotherapy drugs induced apoptosis was confirmed by Annexin-V/PI double staining assay (Fig. 2f). IF and Annexin-V/PI double staining assay confirmed that FZR1 overexpression promotes chemotherapy drugs induced apoptosis in T-47D cells (Supplementary Fig. S2d, e). Furthermore, the apoptosis suppression of FZR1 ko was validated by the molecular markers, cleaved caspase 3 and PARP using IF and western blot (Fig. 2g, h).

Western blot and Annexin-V/PI double staining assay confirmed that overexpression FZR1 promotes chemotherapy drugs induced apoptosis in MBA-MD-231 (Supplementary Fig. S2c, d). Our results suggested that FZR1 can be a potential biomarker of NACT in breast cancer and is involved in the regulation of chemotherapy drugs induced apoptosis.

### FZR1 mediates cisplatin induced apoptosis via p53 stability regulation

**Fig. 3:**
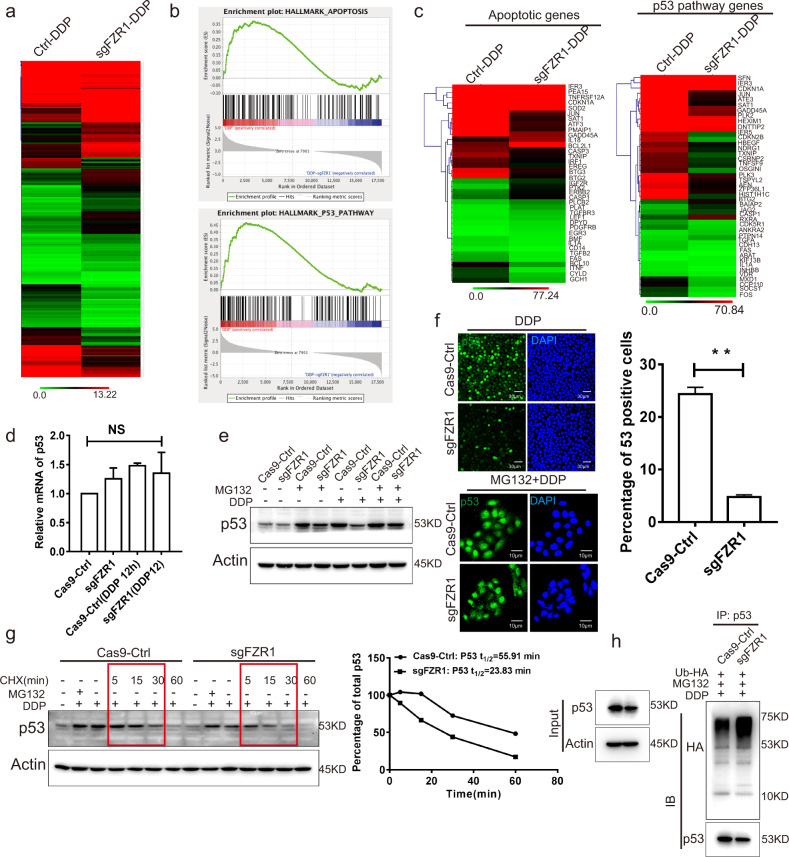
FZR1 promoting chemotherapy drug induced apoptosis by regulating the stability of p53. **a** RNA seq was performed in control and FZR1 ko T-47D cells treated with cisplatin. Heatmap clustered on expression profiles created based on log2 transformed counts to identify consistent changes in various samples. **b** GSEA analysis of apoptosis and p53 pathway relevant gene sets enriched among genes up- and down-regulated in control and FZR1 ko T-47D cells treated with cisplatin. **c** Heatmap clustered on apoptosis and p53 pathway relevant gene sets expression profiles in control and FZR1 ko T-47D cells treated with cisplatin. **d** The mRNA levels of p53 in control and FZR1 ko T-47D cells with or without cisplatin treatment were measured by qPCR. **e** Western blot was performed to evaluate the stability of p53 in control and FZR1 ko T-47D cells treated with cisplatin and proteasome inhibitor MG132. **f** IF of p53 was performed in control and FZR1 ko T-47D cells treated with cisplatin or cisplatin and proteasome inhibitor MG132. A relative quantification of the number of p53 positive cells ± SD of three independent experiments is shown in the histogram on the right. **p < 0.01; scale bar 30 or 10 µm. **g** The protein stability of p53 was evaluated in control and FZR1 ko T-47D cells treated with cisplatin, proteasome inhibitor MG132 and protein synthesis inhibitor CHX for 5, 15, 30, and 60 min. A representative western blot of three independent experiments is shown. **h** IP using p53 antibody was performed in control and FZR1 ko T-47D cells expressing ubiquitin with HA tag treated with cisplatin and proteasome inhibitor MG132. The precipitate was detected by western blot using HA antibody.

Studies are evidenced that many signaling pathways are involved in the chemotherapy drug induced apoptosis^28,29^. To screen the relevant molecules of apoptosis, RNAseq analysis was performed in control and FZR1 ko T-47D cells treated with cisplatin. A subset of approximately 450 significantly differentially expressed genes was identified between control and FZR1 ko T-47D cells (Fig. 3a). The raw data of RNAseq has been submitted to the GEO repository with the accession number GSE142525. GSEA analysis revealed that the hallmark of apoptosis and p53 pathway were significantly enriched in control sample with the positive correlation and in FZR1 ko sample with negative correlation (Fig. 3b).The gene ontology analysis indicated that the significantly differentially expressed genes were enriched in apoptosis and p53 pathways (Fig. 3c). These data indicate that FZR1 is involved in the p53 pathway to mediate chemotherapy drugs induced apoptosis.

To gain insight into the impact of FZR1 on p53 pathway, we evaluated the mRNA levels of p53 in control and FZR1 ko T-47D cells with or without cisplatin treatment. The qPCR data showed that FZR1 ko did not impair the transcription of p53 (Fig. 3d). Furthermore, western blot demonstrated that the protein level of p53 is decreased in FZR1 ko T-47D cells with cisplatin treatment. While the protein level of p53 is recovered in FZR1 ko T-47D cells with proteasome inhibitor MG132 treatment (Fig. 3e). We also confirmed the protein level of p53 is decreased in FZR1 ko T-47D cells with doxorubicin treatment and recovered with proteasome inhibitor MG132 treatment (Fig. S4a). The IF results confirmed that FZR1 ko significant reduced the p53 positive cell population treated with cisplatin. The percentage of p53 positive cell in FZR1 ko was identical to the control while treated with cisplatin and MG132 (Fig. 3f). The p53 was reported with a short half-life and degraded within 0.5 h through ubiquitin pathway^30^. To evaluate the half-life of p53 in control and FZR1 ko T-47D cells, the cells were treated with cisplatin and a eukaryote protein synthesis inhibitor Cycloheximide (CHX), for 5, 15, 30, and 60 min. Western blot result indicated that FZR1 ko obviously decreased the half-life of p53 (Fig. 3g). Furthermore, IP result revealed that FZR1 ko significantly promotes p53 ubiquitination (Fig. 3h).

We also confirmed the expression of DNA damage marker γH2AX in FZR1 overexpression and ko T-47D cells with cisplatin treatment (Supplementary Fig. S4b, c). As a cell cycle regulator, FZR1 is involved in the regulation of mitosis and the G1 phase of the cell cycle. We explored the impact of FZR1 overexpression and ko on cell proliferation. The EDU cell proliferation assay indicated that FZR1 overexpression and ko have not impact on cell proliferation (Supplementary Fig. S4d–f). FZR1 overexpression and ko cells were treated with cisplatin and were performed the cell cycle analysis using FACS and Western blot. The data showed that FZR1 ko promoted T-47D cell division arrest with cisplatin treatment (Supplementary Fig. S5a–c). These results suggest that FZR1 ko promote the resistance of tumor cell to chemotherapeutic drugs by impairing cell cycle regulation.

**Fig. 4:**
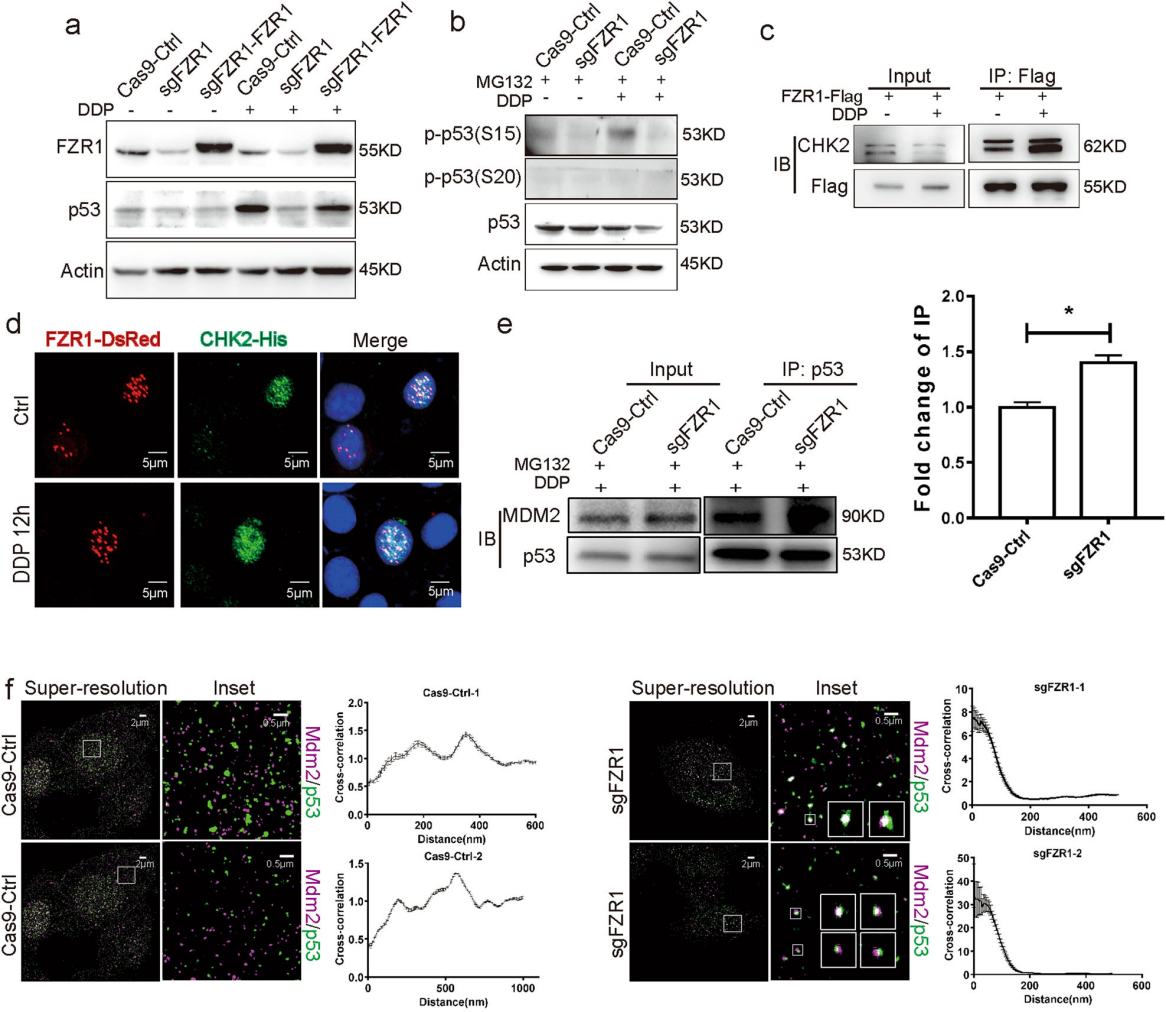
FZR1 mediated p53 stability by interacting with CHK2 and activating MDM2 pathways. **a** FZR1 ko T-47D cells expressed synonymous mutant FZR1 to obtain rescue cells. Western blot was performed in FZR1 ko and rescue T-47D cells to measure the protein levels of FZR1 and P53 with and without cisplatin treatment. **b** The protein levels of phosphorylated p53 was evaluated in control and FZR1 ko T-47D cells treated with cisplatin and MG132. A representative western blot of three independent experiments is shown. **c** IP using Flag antibody was performed in FZR1-Flag overexpressed T-47D cells treated with cisplatin. The precipitate was detected by western blot using CHK2 antibody. **d** IF using His antibody to detected CHK2 was performed in overexpressed FZR1-Dsred and CHK2-His T-47D cells treated with cisplatin; scale bar 5 µm. **e** IP using p53 antibody was performed in control and FZR1 ko T-47D cells treated with cisplatin and MG132. The precipitate was detected by western blot using MDM2 antibody. A relative quantification of the value of gray is shown in the histogram on the right. *p < 0.05. **f** Two-color STORM of p53 and MDM2 was performed in control and FZR1 ko T-47D cells treated with cisplatin and MG132. Calculated two-dimensional cross-correlations between the two channels at different intermolecular distances based on control or ko FZR1. The value of cross-correlation indicated the single molecules of p53 and MDM2 crossed index of each other. The value of distance showed the single molecules of p53 and MDM2 distance distribution in the cell; scale bar 2 or 0.5 µm.

Next, we explored the molecular mechanism of FZR1 impairing the stability of p53. To confirm the impact of FZR1 on p53 degradation, the rescue cells of mutant FZR1 overexpressed in FZR1 ko T-47D cells were generated. Western blot result of control, FZR1 ko, and FZR1 ko rescue T-47D cells showed that p53 stability is significantly increased in rescue cells (Fig. 4a). The stability of p53 is regulated by the sites phosphorylation of Ser15 and Ser20 via protein kinase ATM, ATR, or Chk2, Chk1. The phosphorylated p53 at Ser15 and Ser20 leads to a reduced interaction between p53 and MDM2 that promotes p53 accumulation by inhibiting ubiquitination and proteasomal degradation^31,32^. Western blot result indicated that phosphorylation at Ser15 of p53 was suppressed in FZR1 ko T-47D cells (Fig. 4b). The association of FZR1 and Chk2 was confirmed by IP and IF in FZR1 and Chk2 overexpressed cells (Fig. 4c, d). The interaction between p53 and MDM2 was validated by IP and super-resolution STORM microscopy in control and FZR1 ko cells (Fig. 4e, f). Our data demonstrated that FZR1 is required for regulating the stability of p53 by phosphorylating at Ser15 site.

### In vivo and clinical validation of FZR1 as a potential biomarker for NACT

**Fig. 5:**
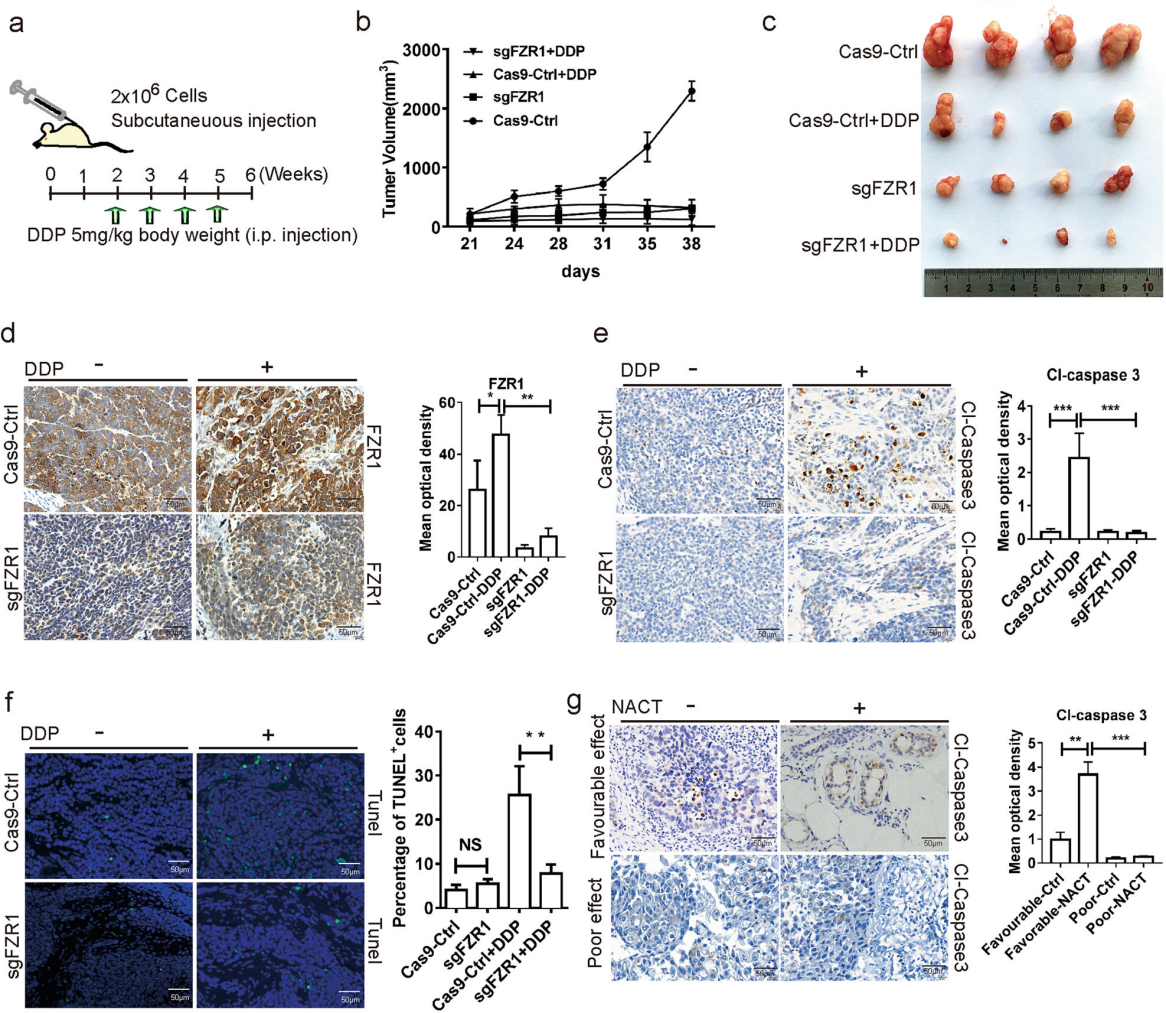
FZR1 mediates chemotherapy drug induced apoptosis in vivo. **a** FZR1 ko and control T-47D cells were injected in subcutaneous xenografts and treated with cisplatin. The flow chart schedule of cell injection and treatment, and the tumor size were measured twice a week in two dimensions throughout the experiments. **b** The tumor volume curves of xenograft post FZR1 ko and control T-47D cells injection. **c** Image of excised FZR1 ko and control T-47D cells xenograft tumors in nude mice. **d** IHC using FZR1 antibody in FZR1 ko and control tumor tissues with or without cisplatin treatment. The quantification is representative of the optical density in 10 fields ±SD by random. *p < 0.05, **p < 0.01; scale bar 50 µm. **e** IHC of cleaved-caspase 3 in FZR1 ko and control tumor tissues with or without cisplatin treatment. The quantification is representative of the optical density in 10 fields ±SD by random. ***p < 0.005; scale bar 50 µm. **f** The apoptosis analysis by TUNEL in FZR1 ko and control tumor tissues with or without cisplatin treatment. The quantification is representative of the percentage of positive cells in 10 fields ± SD by random. **p < 0.01, NS: not significant; scale bar 50 µm. **g** IHC of cleaved-caspase 3 was performed in patient derived tumor tissues prior to or post NACT with poor or favorable effect. The quantification is representative of the optical density in 10 fields ±SD by random. **p < 0.01, ***p < 0.005; scale bar 50 µm.

To confirm the functional role of FZR1, we examined the animal model in vivo. Nude mice xenografts were generated by FZR1 ko and control T-47D cells and treated with cisplatin. The result showed that FZR1 ko and cisplatin treatment significantly reduced tumor growth (Fig. 5a–c). The IHC result indicated that FZR1 ko significantly reduced cleaved caspase 3 positive cells (Fig. 5d, e). The TUNEL assay reveals that the percentage of positive cells significantly decreased in FZR1 ko tumors (Fig. 5f). Furthermore, to validate the functional role of FZR1 induced apoptosis by chemotherapy drugs, we performed the IHC for the patient tumor samples using cleaved caspase 3 antibody. The results demonstrated that cleaved caspase 3 positive cells significantly increased in the favorable effect patient samples with NACT treatment (Fig. 5g). These data demonstrated that FZR1 is required for the chemotherapy drugs induced apoptosis in vivo.

**Fig. 6:**
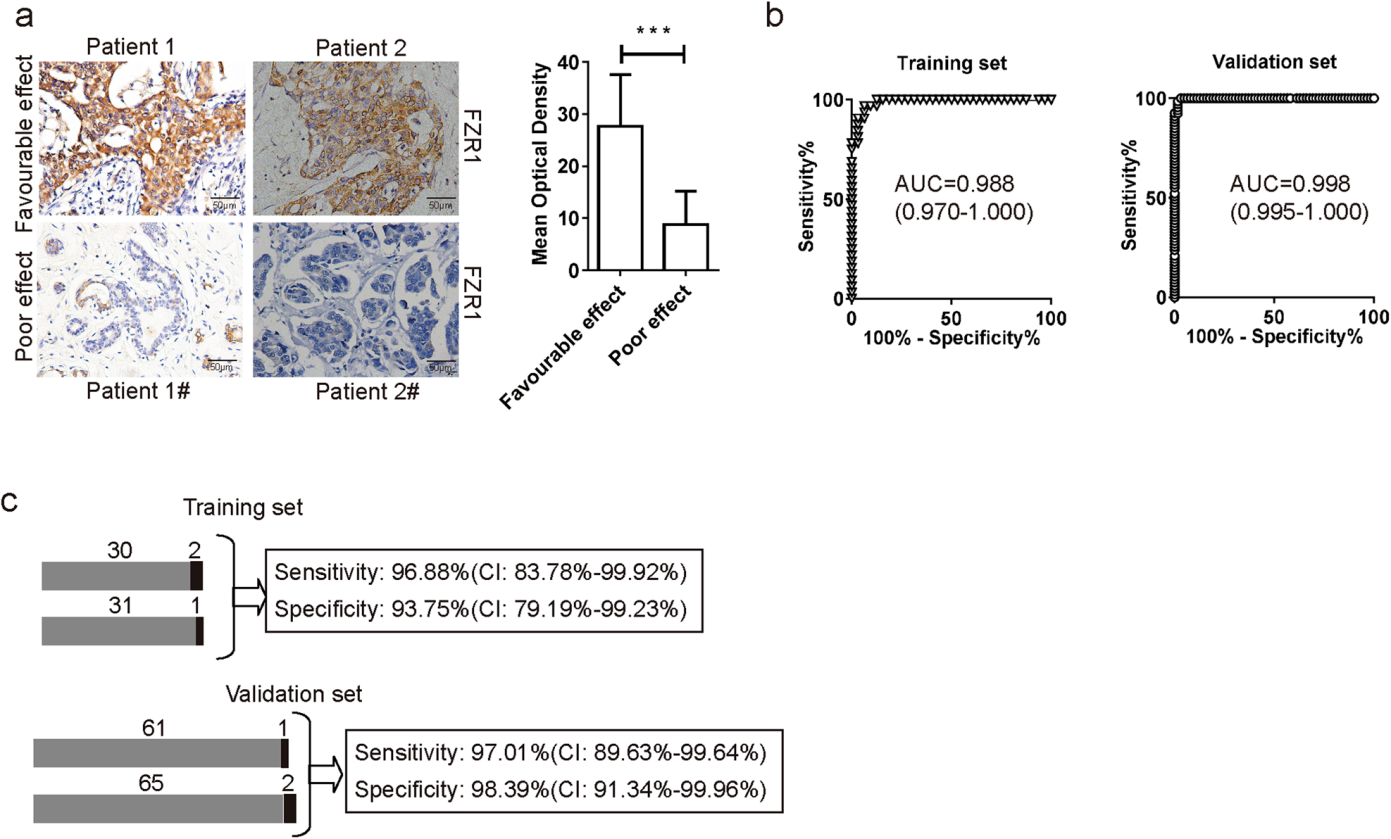
Clinical validation of FZR1 as the biomarker for breast cancer neoadjuvant chemotherapy. **a** IHC of FZR1 in clinical patients samples with clinical affirmed favorable and poor effect of neoadjuvant chemotherapy. The quantification is representative of the optical density in 10 fields ±SD by random. ***p < 0.005; scale bar 50 µm. **b** Clinical patients’ samples were randomly divided (1:2) into training and validation set. ROC curve analysis on the training set was used to determine cut-off points to discriminate the favorable and poor effect of neoadjuvant chemotherapy. The AUC with 95% CI and ROC curves of the validation set under the same parameters are shown. **c** Sensitivity and specificity including the confidence intervals in the validation set for the favorable and poor effect of neoadjuvant chemotherapy are shown for FZR IHC score (cut-off point: FI > 17.5) as the prediction biomarker.

All the clinical samples were performed IHC and quantification using the Optical density score method in the 10 fields by random^33,34^. The data indicated that the expression of FZR1 significantly downregulated in the poor effect of NACT group (Fig. 6a). We divided the patients into a training set (1/3) and a validation set (2/3) by random to define and test cut off point by inspection of ROC curves. The cut off point for the expression of FZR1 with IHC score was 17.5 (Fig. 6b). The statistical evaluation of the training set and validation set revealed a sensitivity of 97.01% (CI: 89.63–99.94%) and a specificity of 98.39% (CI: 91.34–99.96%) for NACT effect prediction using FZR1 biomarker (Fig. 6c). Thus, the complementarity of the expression of FZR1 biomarker strengthens the reliability of NACT effect prediction and statistical evaluation confirmed the stability of the test procedure for future clinical evaluation.

## Discussion

At present, the consensus and standard care for breast cancer should combine neoadjuvant or adjuvant therapy followed by surgical intervention^35^. PCR is the most important favorable prognostic effect related to the better treatment outcome. However, approximately more than half of the patients cannot achieve PCR with NACT^36,37^. To avoid chemotherapy toxicity and surgery delay in non-responders, it is greatly need to identify the biomarker for the efficiency of NACT. There are several studies tried to identify the predictor for the efficiency of chemotherapy. A genomic predictor combining ER status were analyzed to predict the probability of survival of patients following taxane and anthracycline chemotherapy^38^. Paul Gass and colleagues evidenced that the effect of NACT may be greatest in the high grade tumor of patients with triple-negative breast cancer^36^. Here we have taken clinical patients with NACT cohort analysis and the publicly available transcriptomic data of GEO and TCGA screening to identify the biomarker signature for the efficiency of NACT. Our investigation revealed that FZR1 can be a potential biomarker for breast cancer NACT prediction through regulating apoptosis and cell cycle arrest.

Based on the clinical image and diagnosis evaluation, we revealed that the effective NACT or patients obtained significantly benefits is less than 50% within our clinical patients cohort. The validation of the public available dataset GEO and TCGA indicated the consistent result. The dataset of the publicly available transcriptomic data of GEO has been used and proved in a variety of studies^39–42^. Our analysis identified FZR1 as a biomarker of NACT for breast cancer regulating apoptosis and cell cycle arrest. FZR1 is a co-activator of the Anaphase Promoting Complex or Cyclosome (APC/C), which is an E3 ubiquitin ligase regulating mitosis and the G1 phase of the cell cycle^43^. APC/C-FZR1 remains active to G1 phase and is inactivated by phosphorylation at the G1/S transition into S and G2 phase. Ultimately, FZR1 is activated by dephosphorylation with CDK1 inactivation in anaphase of mitosis^44^. In response to DNA damage, APC/C-FZR1 is reactivated to degrade Claspin, which mediated activation of Chk1 in G2 DNA damage response checkpoint^45^. A variety of chemotherapy drugs are targeted to induce cancer cell apoptosis via DNA damage. Our data revealed that FZR1 is required for DNA damage induced Chk2 activation and the phosphorylation of p53 with the chemotherapy drugs treatment. Recently reported that knockdown FZR1 and Rb in human breast cancer cells promote cell division arrest by CDK4/6-specific inhibitor Palbociclib (PD0332991) treatment^46,47^. Our data demonstrated that FZR1 ko promoted T-47D cell division arrest with cisplatin treatment. These results suggest that FZR1 ko affects breast cancer cell resistance to chemotherapeutic agents by regulating cell cycle arrest. In recent work, it is reported that FZR1 inhibits BRAF oncogenic functions via both APC-dependent proteolysis and APC independent disruption of BRAF dimers. FZR1 is considered as a tumor suppressor that are negatively regulated the activation of the MEK/ERK oncogenic signaling cascade^48^. The evidence showed that FZR1 interacted with PRL-3 to regulate the progression of colorectal cancer by controlling the stability of AURKA^49^. These results are consistent with our clinical validation that the expression of FZR1 is correlated with the prognosis and survival of breast cancer patients. FZR1 has been reported as both a tumor suppressor and oncoprotein in a variety of cancer types. Loss of FZR1 contributes to the development of chemotherapy resistant clones in mouse and human B-cell acute leukemia^50^. FZR1 inhibits the replicative stress and p53-dependent cell death in neural progenitors. These data are consistent with our analysis that FZR1 modulated apoptosis via p53 stability. The biomarker to display the efficiency of NACT that is reasonable to involve in apoptosis and cell cycle arrest. The protein level of FZR1 contributes to the apoptosis induction of chemotherapy drugs. Taken together, our results demonstrated that FZR1 can be a potential biomarker for NACT in breast cancer.

IHC staining is a powerful method to identify the expression of specific antigens in formalin-fixed, paraffin embedded tissues, which is widely used for diagnostics in clinical and research field. The Optical density score method was established and used widely to quantify the IHC samples. The FZR1 IHC score of individual patients was calculated and statistical evaluation by ROC curve. The training set was use to define the cutoff point at 17.5 with 1 false positive in poor effect patient group and 2 false negative in favorable effect patient group. The IHC score at 17.5 was validated by the validation set with 2 false positive in poor effect patient group and 1 false negative in favorable effect patient group. The expression of FZR1 for NACT effect prediction reach a sensitivity of 97.01% (CI: 89.63–99.94%) and a specificity of 98.39% (CI: 91.34–99.96%). These data demonstrated that FZR1 is an efficient biomarker for NACT effect prediction. The FZR1 IHC score can be used at the clinic to evaluate the effect of NACT, which will provide a better therapy for patient.

## Conclusion

This study investigated the functional role of FZR1 on chemotherapy drugs induced apoptosis in vitro and in vivo. The results demonstrated that FZR1 is involved in the stability of p53 by impairing the phosphorylation at ser15 site. It is a significant sensor of the p53 dependent apoptosis on the effect of chemotherapy. The clinical data and the IHC of FZR1 expression proved that FZR1 can be an effective predictor of neoadjuvant chemotherapy in clinical patient cohort.

## Supplementary information

Supplementary Tables

Supplementary figure legends

Figure S1

Figure S2

Figure S3

Figure S4

Figure S5

## Data Availability

The detailed procedures of methods, six figures, one table, three supplementary tables and three supplementary figures are attached.
