# Supplementary material for: FZR1 as a novel biomarker for breast cancer neoadjuvant chemotherapy prediction
Source: Cell Death Dis. 2020 Sep 25;11(9):804. doi: 10.1038/s41419-020-03004-9 (PMC7519164; doi:10.1038/s41419-020-03004-9)
Supplement: Supplementary file 1 — Supplementary Tables [file 41419_2020_3004_MOESM1_ESM.docx]

**Supplementary Tables**

**Supplementary Table 1. Primers for gene overexpression, shRNAs for gene knockdown and sgRNA for gene knockout**

| Gene | Primer | Sequences (5’-3’) |
| --- | --- | --- |
| FZR1-flag-pLV | F | GCTCTAGAGCCACCATGGACCAGGACTATGA |
|  | R | CGGCTAGCTCACTTATCGTCGTCATCCTTGTAATCCCGGATCCTGGTGAAGAGGTTG |
| CHK2-His-pLV | F | GCTCTAGAGCCACCATGTCTCGGGAGTCGGATGTT |
|  | R | CTAGCTAGCTCAATGGTGATGGTGATGATGCAACACAGCAGCACACACAGCT |
| FZR1-mutant-pLV | F | GCAGATCGTTGCATACGTTTTTGGAACACGCTGACAGGACA |
|  | R | AAAACGTATGCAACGATCTGCTGTGCCGCCCCCCGAGGCCA |
| FZR1-sgRNA1 | F | CACCGGAAGCGGTCTCCGTGCTTGC |
|  | R | AAACGCAAGCACGGAGACCGCTTCC |
| FZR1-sgRNA2 | F | CACCGCTGACCGCTGTATCCGCTTC |
|  | R | AAACGAAGCGGATACAGCGGTCAGC |
| FZR1-sgRNA3 | F | CACCGGATCATGCGGTCGCGGCTCC |
|  | R | AAACGGAGCCGCGACCGCATGATCC |
| FZR1-sgRNA4 | F | CACCGACCACTGCAGTGTATCGACA |
|  | R | AAACTGTCGATACACTGCAGTGGTC |

**Supplementary Table 2. Primers for qPCR**

| Gene | Primer | Sequences (5’-3’) |
| --- | --- | --- |
| Bcl-2 | F | CTTTGAGTTCGGTGGGGTCA |
|  | R | GAAATCAAACAGAGGCCGCA |
| Bax | F | CCCGAGAGGTCTTTTTCCGAG |
|  | R | CCAGCCCATGATGGTTCTGAT |
| P53 | F | CAGCACATGACGGAGGTTGT |
|  | R | TCATCCAAATACTCCACACGC |
| GAPDH | F | ACAACTTTGGTATCGTGGAAGG |
|  | R | GCCATCACGCCACAGTTTC |

**Supplementary Table 3. The information of antibodies for WB, IP, IF, IHC.**

| **Name** | **Company** | **Cat. No.** |
| --- | --- | --- |
| TNF-RI | Santa Cruz | SC-7895 |
| TNF-RII | Santa Cruz | SC-7862 |
| Actin | ZSGB-BIO | TA-09 |
| IKB(α) | Cell Signaling Technology | 9242 |
| P-IKB(α) | Cell Signaling Technology | 9246S |
| NF-KB | Santa Cruz | SC-8008 |
| P-NF-KB | Santa Cruz | SC-33020 |
| FZR1 | Abcam | ab89535 |
| Cleaved-caspase3 | Cell Signaling Technology | 9661L |
| CHK2 | Cell Signaling Technology | 0908 |
| MDM2 | Cell Signaling Technology | 86934S |
| γH2AX | Cell Signaling Technology | 9718S |
| P53-total | Santa Cruz | 6243 |
| PARP | ONCOGENE | AM30 |
| P-P53(S15) | Cell Signaling Technology | 9284T |
| P-P53(S20) | Cell Signaling Technology | 9287T |
| P-P53(S392) | Cell Signaling Technology | 9281 |
| Flag-M | Sigma | F1804 |
| Flag-R | Cell Signaling Technology | 14793S |
| HA | Sigma | H3663 |
| C-myc | Santa Cruz | SC-40 |
| His | Santa Cruz | SC-8036 |
| Cyclin D1 | Cell Signaling Technology | 2922S |
| Cyclin B1 | Cell Signaling Technology | 4138 |
| Cyclin E1 | Cell Signaling Technology | 20808 |
| CDC20 | Cell Signaling Technology | 77055S |
| P-CDC20(Y15) | Cell Signaling Technology | 9111S |
| P-IKK-(R) | Cell Signaling Technology | 2687 |
