# Supplementary material for: FZR1 as a novel biomarker for breast cancer neoadjuvant chemotherapy prediction
Source: Cell Death Dis. 2020 Sep 25;11(9):804. doi: 10.1038/s41419-020-03004-9 (PMC7519164; doi:10.1038/s41419-020-03004-9)
Supplement: Supplementary file 2 — Supplementary figure legends [file 41419_2020_3004_MOESM2_ESM.docx]

**Supplementary figure legends**

**Fig. S1 Survival curve of candidate genes for neoadjuvant chemotherapy effect prediction**

The survival curve of 12 candidate genes that was analyzed by the online tool of KMPLOT.

**Fig. S2 Chemotherapeutic drugs promote FZR1 expression and involved in apoptosis**

(a) mRNA levels of FZR1 were detected by qPCR in T-47D cells treated with cisplatin for various time as showing. The relative expression of FZR1 was calculated using the ΔCt method and GAPDH as normalization control. The graph represents the average of FZR1 relative expression ± SD of 3 times. **p<0.01, ***p<0.005, ****p<0.001. (b) Western blot analysis of FZR1, PARP, Cleaved-caspas 3 and actin expression in T-47D cells treated with cisplatin for various time as showing. (c)Western blot analysis of FZR1, PARP, Cleaved-caspas 3 and actin expression in T-47D cells treated with etoposide for various time and concentrations as showing. (d) Annexin-V/PI double staining was performed in control and FZR1 overexpression T-47D cells treated with cisplatin. The quantification is representative of experiments in triplicate, and the percentages of apoptotic cells are shown in the relevant quadrants. **p<0.01.

**Fig. S3 FZR1 overexpression in MDA-MB-231 cells promote chemotherapeutic drugs induced apoptosis.**

(a) FZR1 fuse Flag tag overexpression MDA-MB-231 stable cell line was generated by lentiviral transduction. (b) FZR1 overexpression decreased the cell viability of MDA-MB-231 to the chemotherapy drug cisplatin treatment with dose and time dependent. The graph represents the average of cell staining absorbance ± SD of 3 different times. *p<0.05, **p<0.01, ***p<0.005. (c) Annexin-V/PI double staining was performed in control and FZR1 overexpression MDA-MB-231 cells treated with cisplatin. The quantification is representative of experiments in triplicate, and the percentages of apoptotic cells are shown in the relevant quadrants. ****p<0.001, NS: not significant. (d) IF of Cleaved-caspase 3 was performed in control and FZR1 overexpressed MDA-MB-231 cells treated with 2 µg/ml cisplatin for 24 h. The quantification are representative of the 10 fields ± SD by random. *p<0.05; scale bar 100 µm. (e) FZR1 overexpression MDA-MB-231 increased the cleavage of apoptotic protein PARP and caspase 3 level with the chemotherapy drugs cisplatin and epirubicin treatment.

**Fig. S4 FZR1 overexpression or knockout do not impair proliferation but impacted the drug induced apoptosis.**

(a) The total protein levels of p53 in control and FZR1 ko T-47D cells treated with doxorubicin and MG132 treatment were tested by western blot. (e) Western blot was performed to evaluate FZR1, γH2AX, actin in control, FZR1 overexpression, FZR1 ko T-47D cells treated with cisplatin for various time points. (c) IF of γH2AX was performed in control, FZR1 overexpression, FZR1 ko T-47D cells treated with cisplatin for 12 hours. The quantification are representative of the 10 fields ± SD by random. *p<0.05; scale bar 30 µm. (d-f) EdU cell proliferation assay was performed to evaluate the impact of overexpression of FZR1, FZR1 ko, and FZR1 ko rescue on T-47D cell division; scale bar 30 µm.

**Fig. S5 FZR1 deficient promotes chemotherapy drug induced cell cycle arrest**

(a-b) Cell cycle analysis was performed by PI staining using FACS in FZR1 overexpressed or ko and control T-47D cells with cisplatin treatment. The quantification is representative of experiments in triplicate, and the percentages of various cell cycle phases are shown. ***p<0.005, ****p<0.001. (c) The biomarkers of cell cycle were tested by western blot in FZR1 overexpressed or ko and control T-47D cells treated with cisplatin.
