# Supplementary figures and images for: FZR1 as a novel biomarker for breast cancer neoadjuvant chemotherapy prediction
Source: Cell Death Dis. 2020 Sep 25;11(9):804. doi: 10.1038/s41419-020-03004-9 (PMC7519164; doi:10.1038/s41419-020-03004-9)

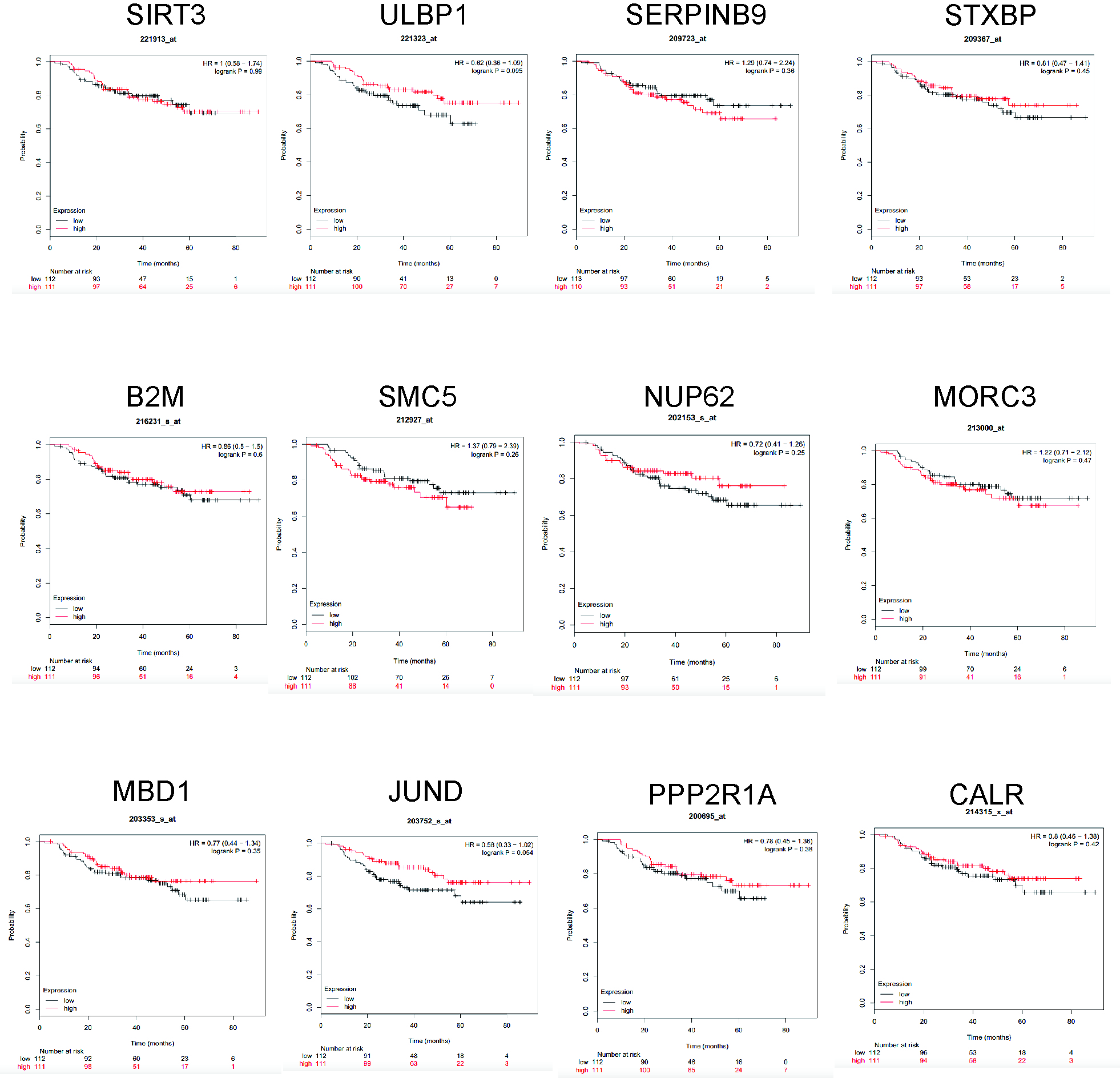

Supplement: Supplementary file 3 — Figure S1 [file 41419_2020_3004_MOESM3_ESM.jpg]

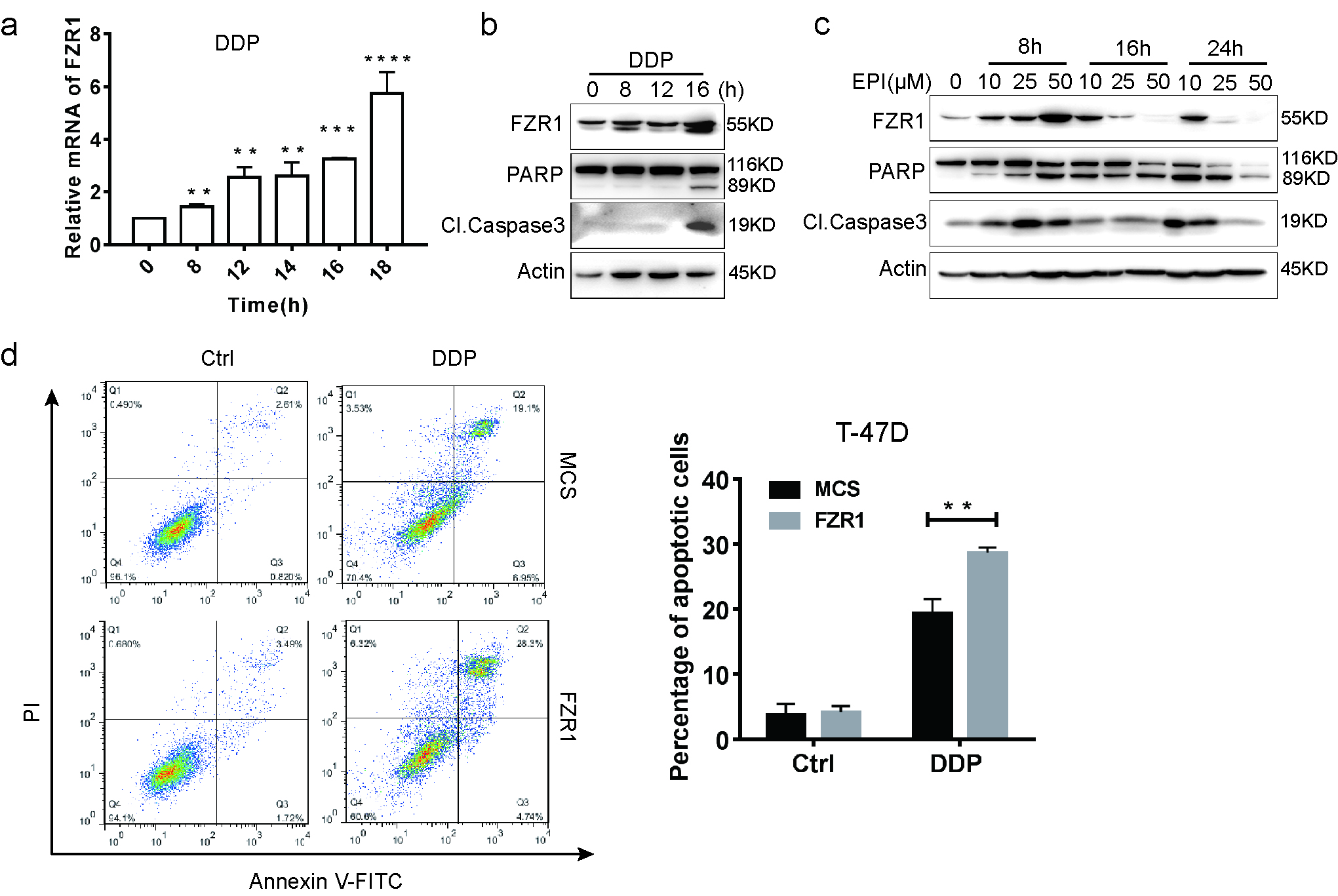

Supplement: Supplementary file 4 — Figure S2 [file 41419_2020_3004_MOESM4_ESM.jpg]

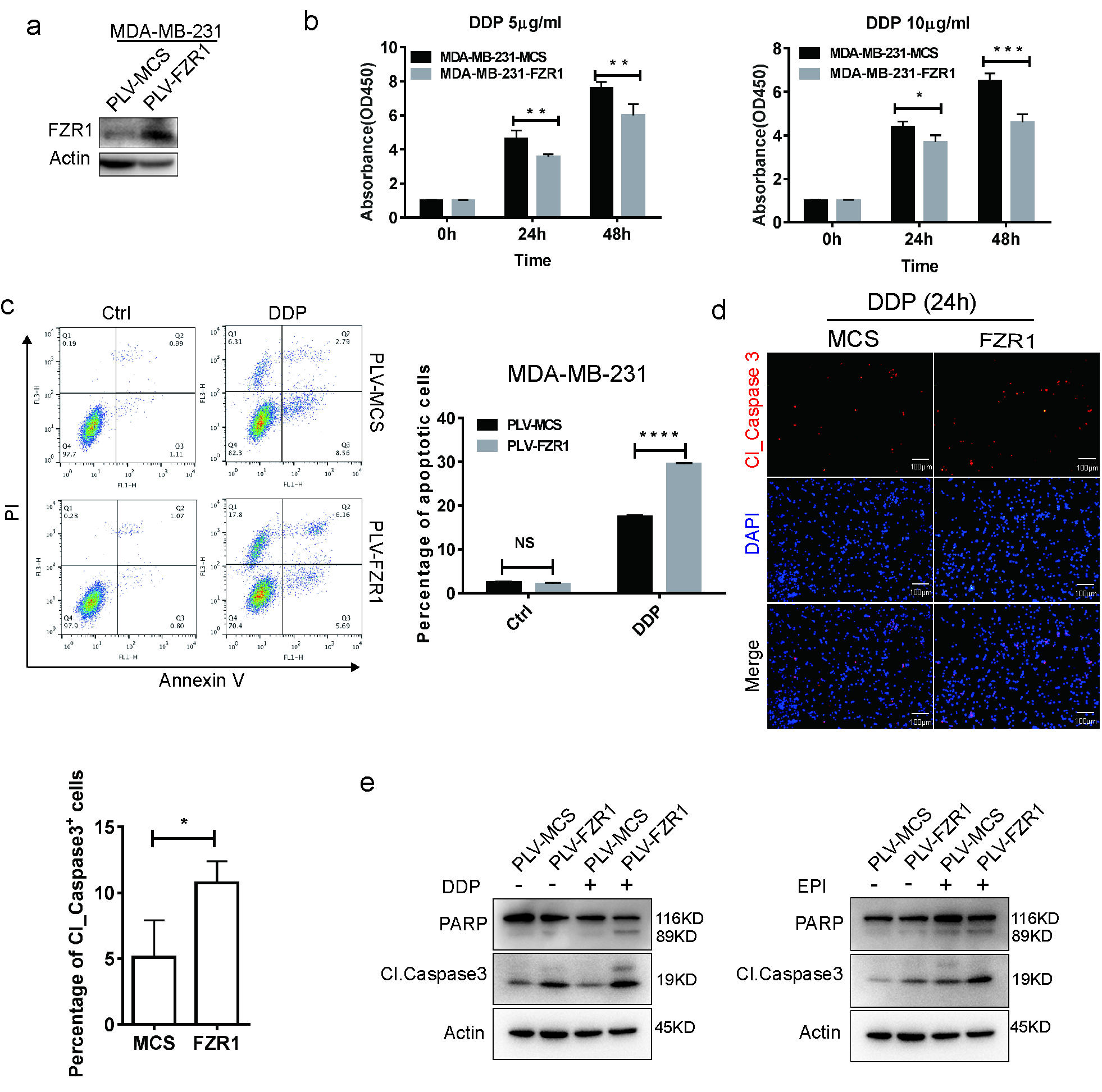

Supplement: Supplementary file 5 — Figure S3 [file 41419_2020_3004_MOESM5_ESM.jpg]

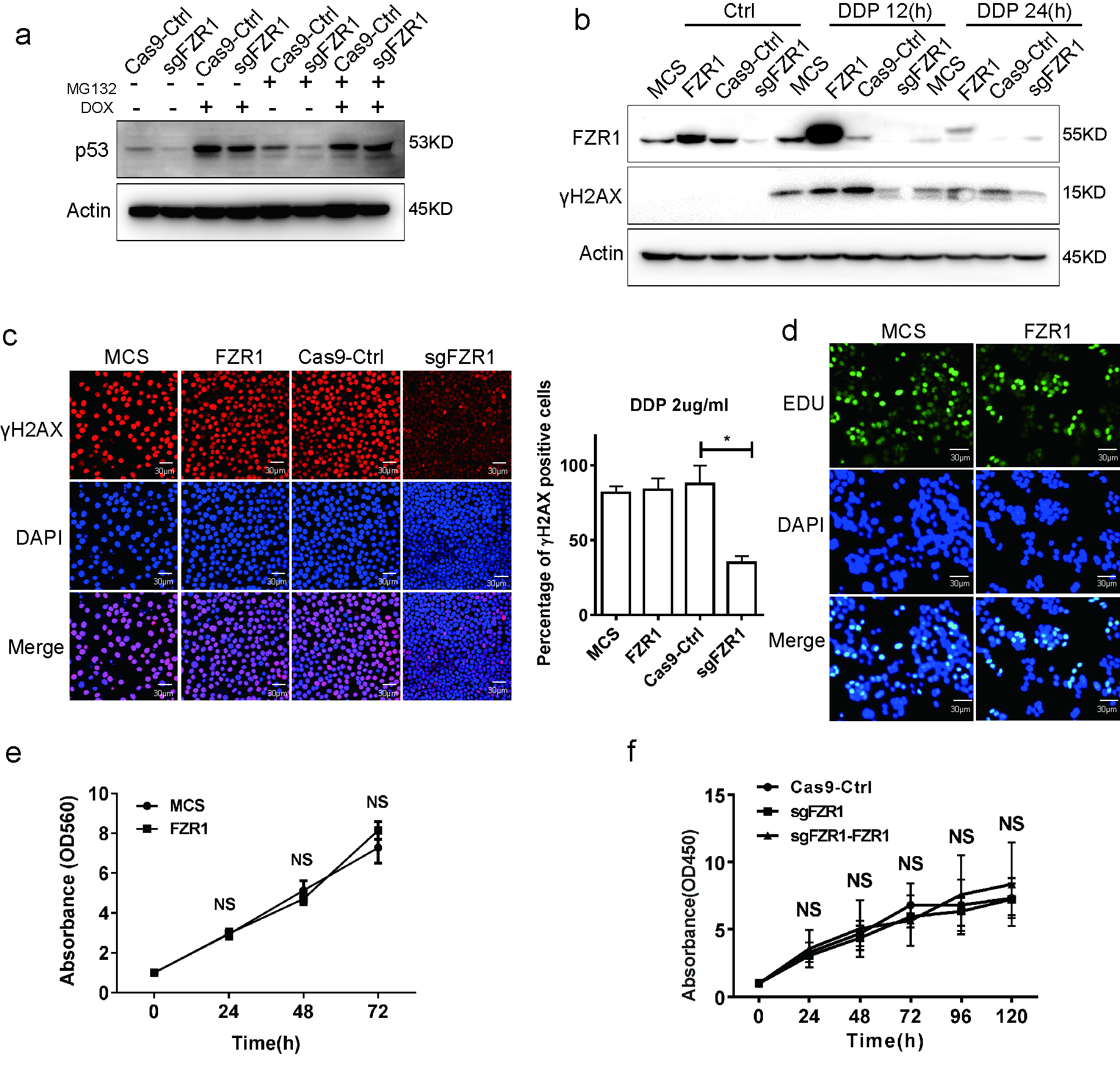

Supplement: Supplementary file 6 — Figure S4 [file 41419_2020_3004_MOESM6_ESM.jpg]

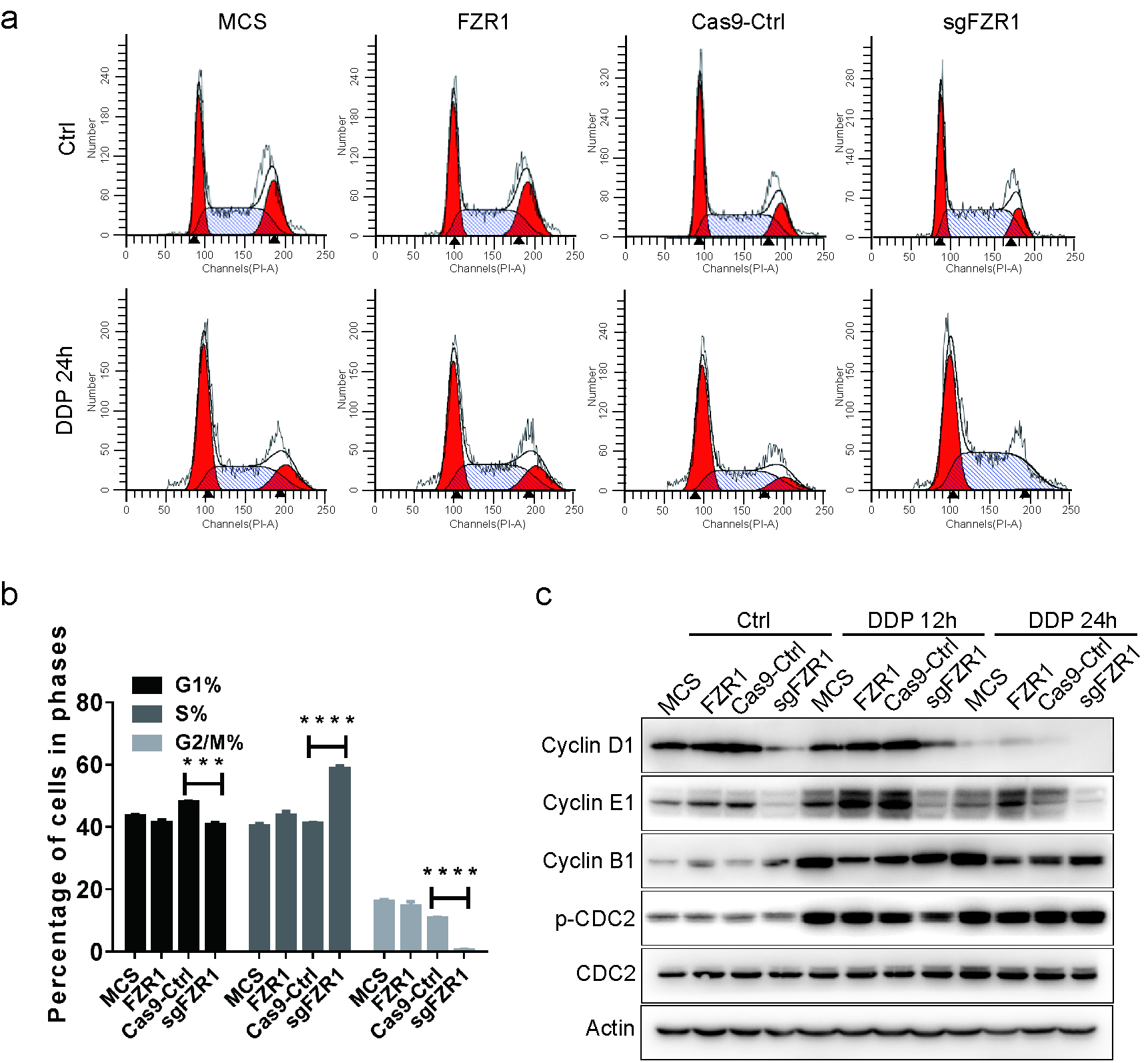

Supplement: Supplementary file 7 — Figure S5 [file 41419_2020_3004_MOESM7_ESM.jpg]
